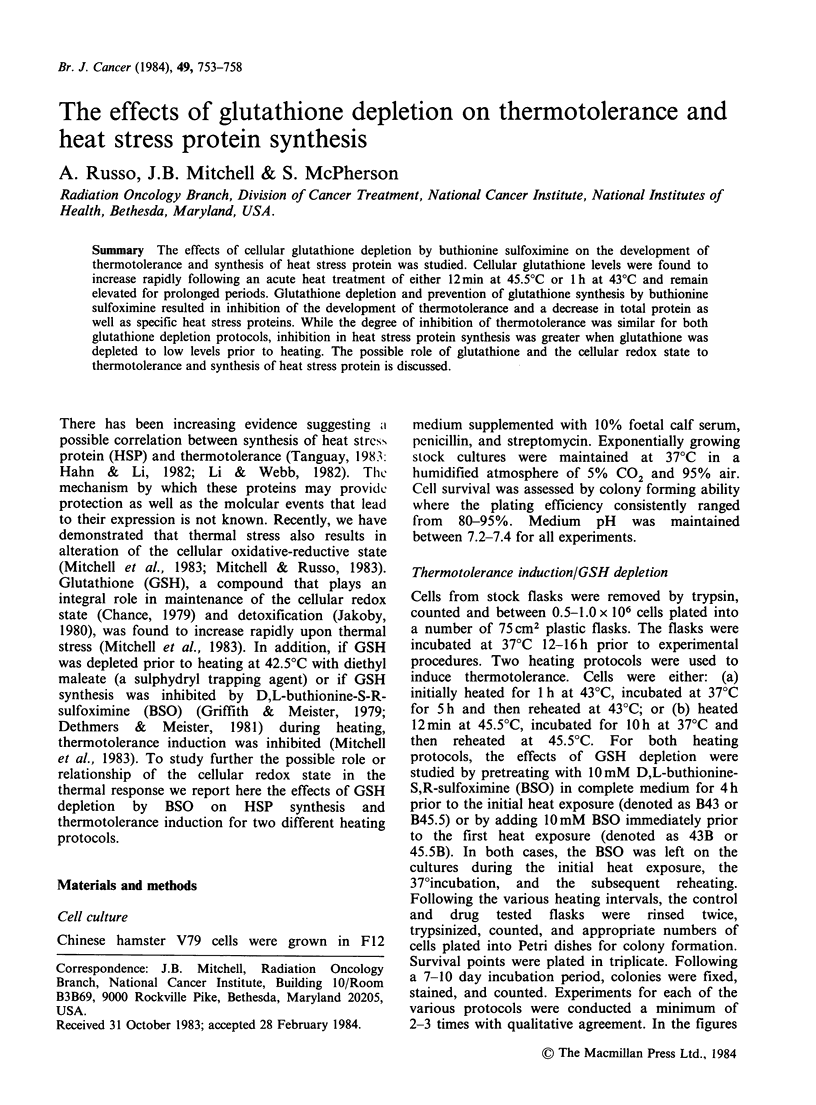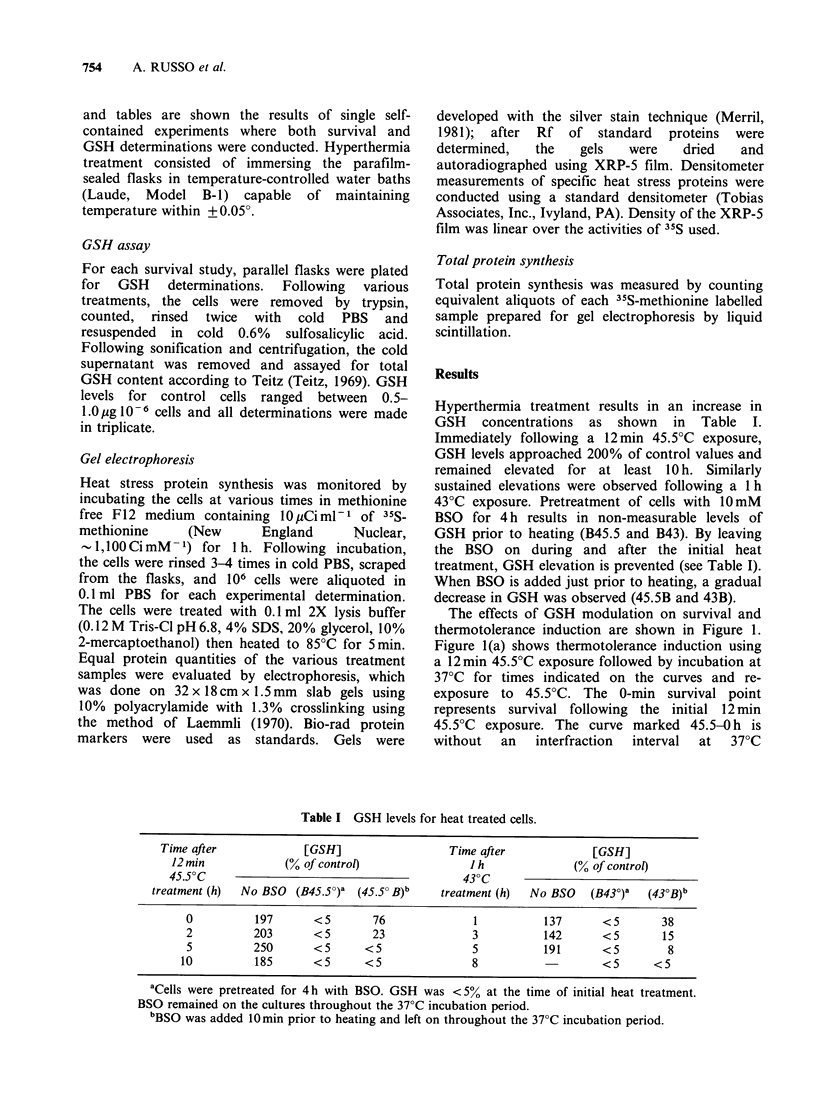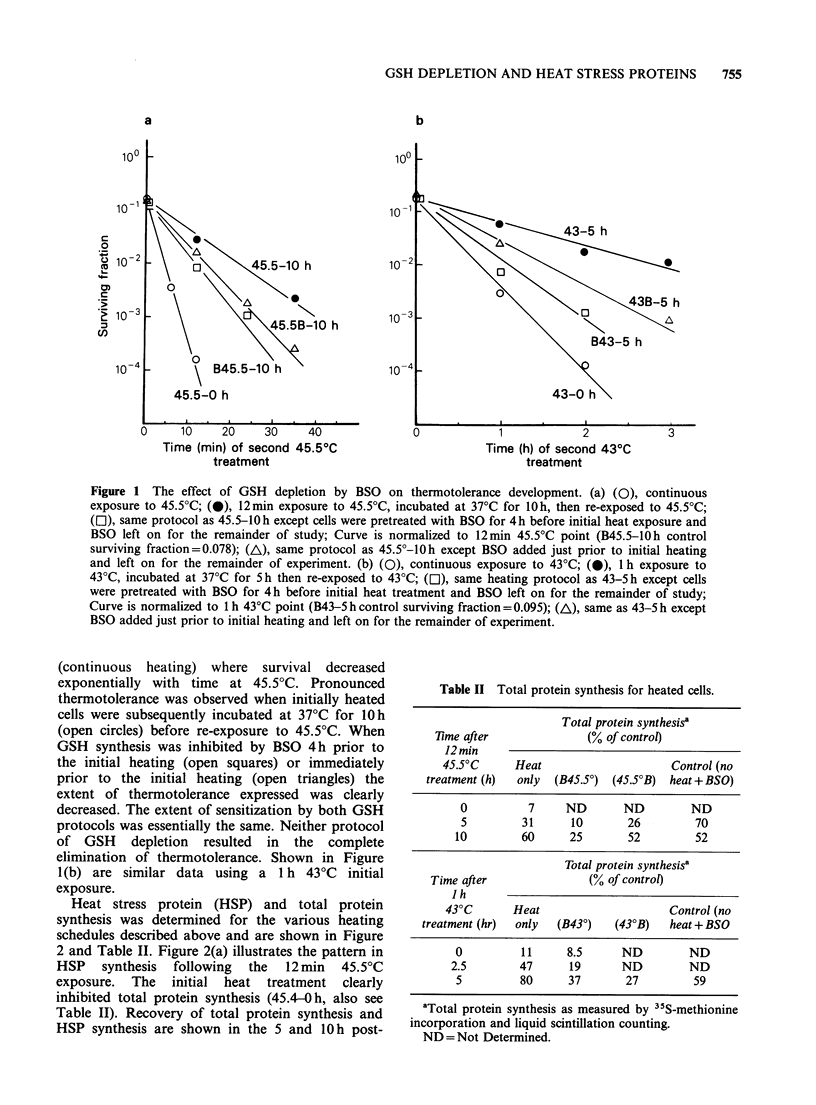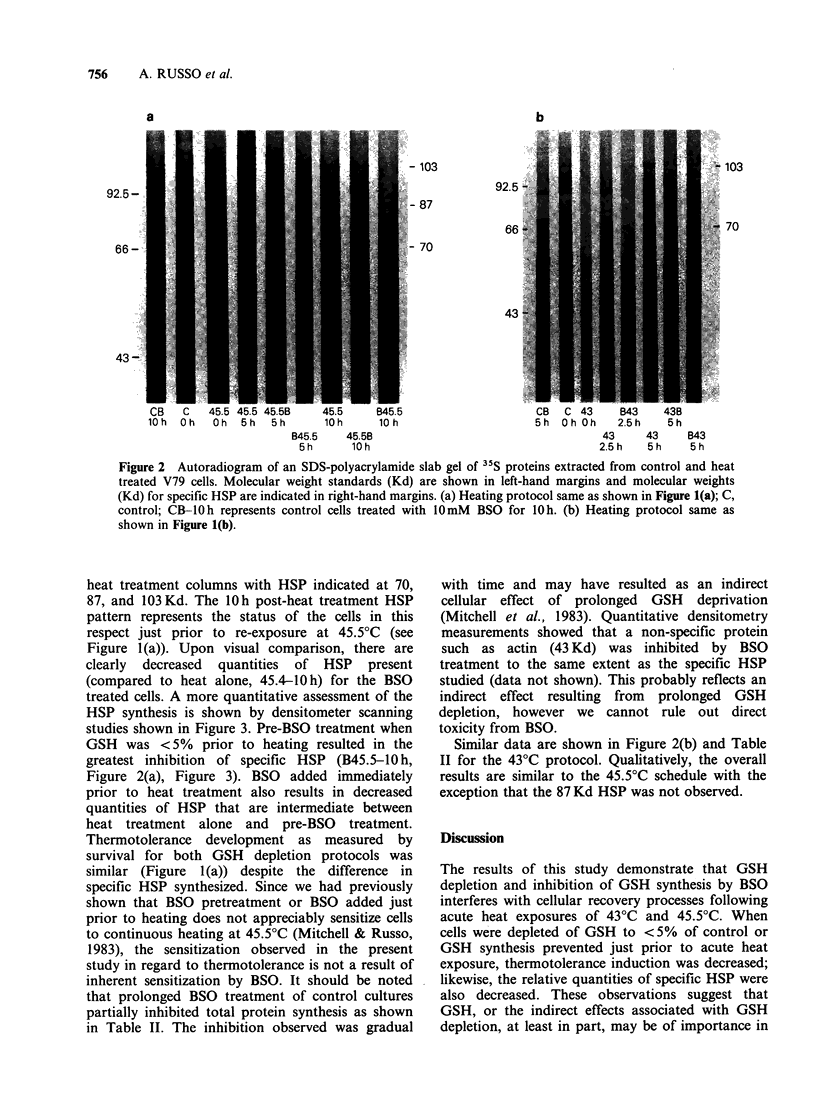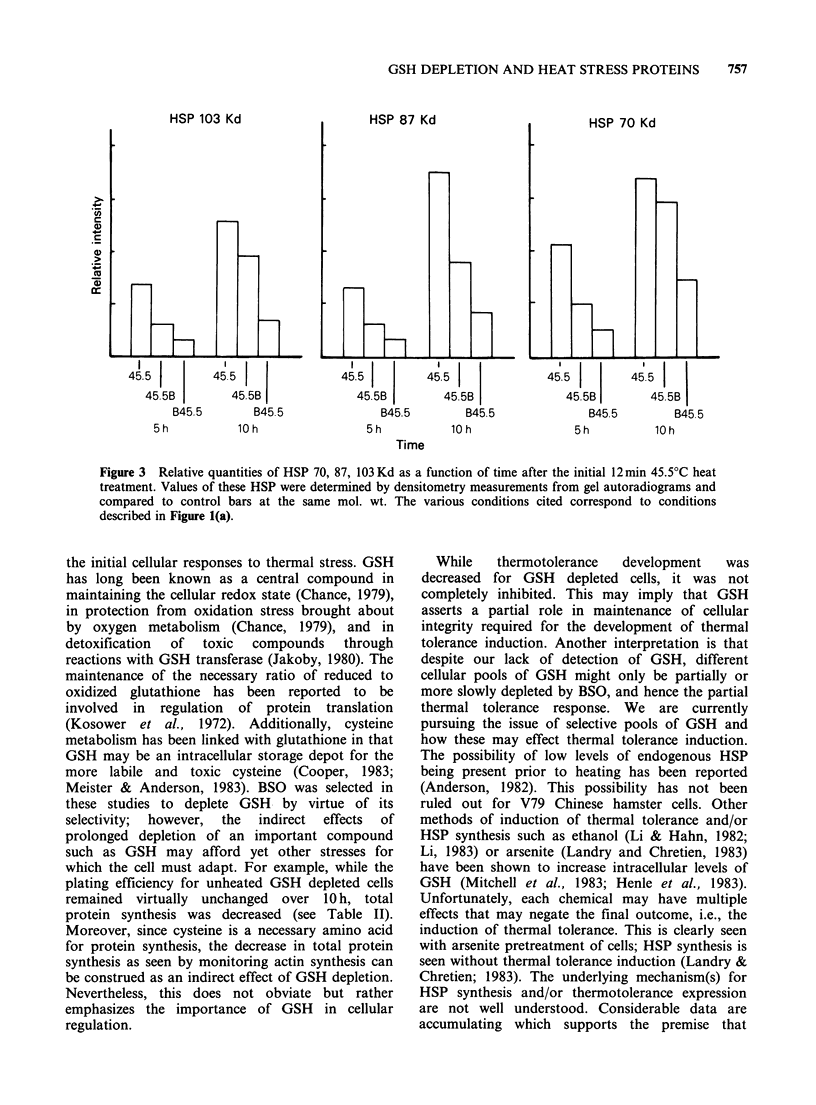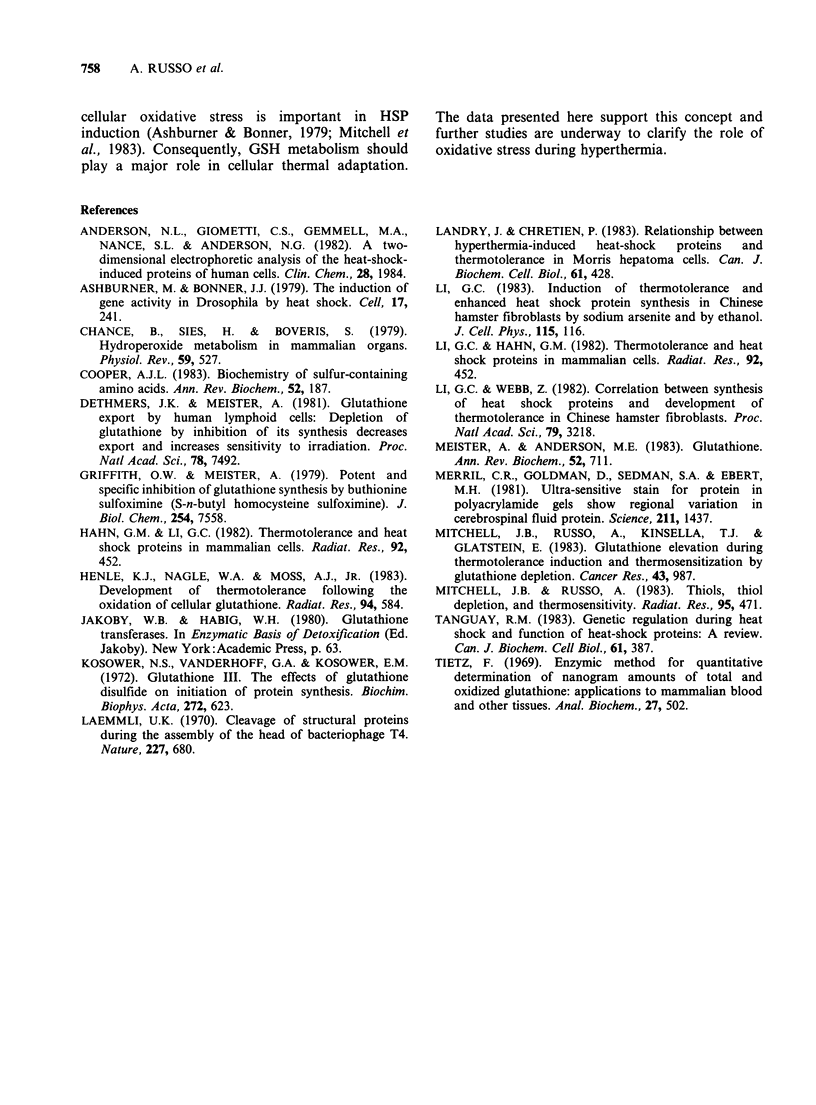# The effects of glutathione depletion on thermotolerance and heat stress protein synthesis.

**DOI:** 10.1038/bjc.1984.118

**Published:** 1984-06

**Authors:** A. Russo, J. B. Mitchell, S. McPherson

## Abstract

**Images:**


*Br. J. Cancer* (1984), **49**, 753–758

### The effects of glutathione depletion on thermotolerance and heat stress protein synthesis

A. Russo, J.B. Mitchell & S. McPherson

*Radiation Oncology Branch, Division of Cancer Treatment, National Cancer Institute, National Institutes of Health, Bethesda, Maryland, USA.*

**Summary** The effects of cellular glutathione depletion by buthionine sulfoximine on the development of thermotolerance and synthesis of heat stress protein was studied. Cellular glutathione levels were found to increase rapidly following an acute heat treatment of either 12 min at 45.5°C or 1 h at 43°C and remain elevated for prolonged periods. Glutathione depletion and prevention of glutathione synthesis by buthionine sulfoximine resulted in inhibition of the development of thermotolerance and a decrease in total protein as well as specific heat stress proteins. While the degree of inhibition of thermotolerance was similar for both glutathione depletion protocols, inhibition in heat stress protein synthesis was greater when glutathione was depleted to low levels prior to heating. The possible role of glutathione and the cellular redox state to thermotolerance and synthesis of heat stress protein is discussed.

There has been increasing evidence suggesting a possible correlation between synthesis of heat stress protein (HSP) and thermotolerance (Tanguay, 1983; Hahn & Li, 1982; Li & Webb, 1982). The mechanism by which these proteins may provide protection as well as the molcular events that lead to their expression is not known. Recently, we have demonstrated that thermal stress also results in alteration of the cellular oxidative-reductive state (Mitchell *et al.*, 1983; Mitchell & Russo, 1983). Glutathione (GSH), a compound that plays an integral role in maintenance of the cellular redox state (Chance, 1979) and detoxification (Jakoby, 1980), was found to increase rapidly upon thermal stress (Mitchell *et al.*, 1983). In addition, if GSH was depleted prior to heating at 42.5°C with diethyl maleate (a sulphydryl trapping agent) or if GSH synthesis was inhibited by D,L-buthionine-S-R-sulfoximine (BSO) (Griffith & Meister, 1979; Dethmers & Meister, 1981) during heating, thermotolerance induction was inhibited (Mitchell *et al.*, 1983). To study further the possible role or relationship of the cellular redox state in the thermal response we report here the effects of GSH depletion by BSO on HSP synthesis and thermotolerance induction for two different heating protocols.

### Materials and methods

### *Cell culture*

Chinese hamster V79 cells were grown in F12 medium supplemented with 10% foetal calf serum, penicillin, and streptomycin. Exponentially growing stock cultures were maintained at 37°C in a humidified atmosphere of 5% $CO_2$ and 95% air. Cell survival was assessed by colony forming ability where the plating efficiency consistently ranged from 80–95%. Medium pH was maintained between 7.2–7.4 for all experiments.

### *Thermotolerance induction/GSH depletion*

Cells from stock flasks were removed by trypsin, counted and between $0.5–1.0 \times 10^6$ cells plated into a number of 75 $cm^2$ plastic flasks. The flasks were incubated at 37°C 12–16 h prior to experimental procedures. Two heating protocols were used to induce thermotolerance. Cells were either: (a) initially heated for 1 h at 43°C, incubated at 37°C for 5 h and then reheated at 43°C; or (b) heated 12 min at 45.5°C, incubated for 10 h at 37°C and then reheated at 45.5°C. For both heating protocols, the effects of GSH depletion were studied by pretreating with 10 mM D,L-buthionine-S,R-sulfoximine (BSO) in complete medium for 4 h prior to the initial heat exposure (denoted as B43 or B45.5) or by adding 10 mM BSO immediately prior to the first heat exposure (denoted as 43B or 45.5B). In both cases, the BSO was left on the cultures during the initial heat exposure, the 37°incubation, and the subsequent reheating. Following the various heating intervals, the control and drug tested flasks were rinsed twice, trypsinized, counted, and appropriate numbers of cells plated into Petri dishes for colony formation. Survival points were plated in triplicate. Following a 7–10 day incubation period, colonies were fixed, stained, and counted. Experiments for each of the various protocols were conducted a minimum of 2–3 times with qualitative agreement. In the figures

Correspondence: J.B. Mitchell, Radiation Oncology Branch, National Cancer Institute, Building 10/Room B3B69, 9000 Rockville Pike, Bethesda, Maryland 20205, USA.
Received 31 October 1983; accepted 28 February 1984.

© The Macmillan Press Ltd., 1984

and tables are shown the results of single self-contained experiments where both survival and GSH determinations were conducted. Hyperthermia treatment consisted of immersing the parafilm-sealed flasks in temperature-controlled water baths (Laude, Model B-1) capable of maintaining temperature within ±0.05°.

*GSH assay*

For each survival study, parallel flasks were plated for GSH determinations. Following various treatments, the cells were removed by trypsin, counted, rinsed twice with cold PBS and resuspended in cold 0.6% sulfosalicylic acid. Following sonification and centrifugation, the cold supernatant was removed and assayed for total GSH content according to Teitz (Teitz, 1969). GSH levels for control cells ranged between 0.5–1.0 $\mu g\, 10^{-6}$ cells and all determinations were made in triplicate.

*Gel electrophoresis*

Heat stress protein synthesis was monitored by incubating the cells at various times in methionine free F12 medium containing 10 $\mu Ci\, ml^{-1}$ of $^{35}S$-methionine (New England Nuclear, ~1,100 $Ci\, mM^{-1}$) for 1 h. Following incubation, the cells were rinsed 3–4 times in cold PBS, scraped from the flasks, and $10^6$ cells were aliquoted in 0.1 ml PBS for each experimental determination. The cells were treated with 0.1 ml 2X lysis buffer (0.12 M Tris-Cl pH 6.8, 4% SDS, 20% glycerol, 10% 2-mercaptoethanol) then heated to 85°C for 5 min. Equal protein quantities of the various treatment samples were evaluated by electrophoresis, which was done on 32 × 18 cm × 1.5 mm slab gels using 10% polyacrylamide with 1.3% crosslinking using the method of Laemmli (1970). Bio-rad protein markers were used as standards. Gels were developed with the silver stain technique (Merril, 1981); after Rf of standard proteins were determined, the gels were dried and autoradiographed using XRP-5 film. Densitometer measurements of specific heat stress proteins were conducted using a standard densitometer (Tobias Associates, Inc., Ivyland, PA). Density of the XRP-5 film was linear over the activities of $^{35}S$ used.

*Total protein synthesis*

Total protein synthesis was measured by counting equivalent aliquots of each $^{35}S$-methionine labelled sample prepared for gel electrophoresis by liquid scintillation.

### Results

Hyperthermia treatment results in an increase in GSH concentrations as shown in Table I. Immediately following a 12 min 45.5°C exposure, GSH levels approached 200% of control values and remained elevated for at least 10 h. Similarly sustained elevations were observed following a 1 h 43°C exposure. Pretreatment of cells with 10 mM BSO for 4 h results in non-measurable levels of GSH prior to heating (B45.5 and B43). By leaving the BSO on during and after the initial heat treatment, GSH elevation is prevented (see Table I). When BSO is added just prior to heating, a gradual decrease in GSH was observed (45.5B and 43B).

The effects of GSH modulation on survival and thermotolerance induction are shown in Figure 1. Figure 1(a) shows thermotolerance induction using a 12 min 45.5°C exposure followed by incubation at 37°C for times indicated on the curves and re-exposure to 45.5°C. The 0-min survival point represents survival following the initial 12 min 45.5°C exposure. The curve marked 45.5–0 h is without an interfraction interval at 37°C

**Table I** GSH levels for heat treated cells.

| *Time after 12 min 45.5°C treatment (h)* | *[GSH] (% of control)* No BSO | *(B45.5°)*[a] | *(45.5° B)*[b] | *Time after 1 h 43°C treatment (h)* | *[GSH] (% of control)* No BSO | *(B43°)*[a] | *(43°B)*[b] |
|---|---|---|---|---|---|---|---|
| 0 | 197 | <5 | 76 | 1 | 137 | <5 | 38 |
| 2 | 203 | <5 | 23 | 3 | 142 | <5 | 15 |
| 5 | 250 | <5 | <5 | 5 | 191 | <5 | 8 |
| 10 | 185 | <5 | <5 | 8 | — | <5 | <5 |

[a]Cells were pretreated for 4 h with BSO. GSH was <5% at the time of initial heat treatment. BSO remained on the cultures throughout the 37°C incubation period.
[b]BSO was added 10 min prior to heating and left on throughout the 37°C incubation period.

**Figure 1** The effect of GSH depletion by BSO on thermotolerance development. (a) (○), continuous exposure to 45.5°C; (●), 12 min exposure to 45.5°C, incubated at 37°C for 10 h, then re-exposed to 45.5°C; (□), same protocol as 45.5–10 h except cells were pretreated with BSO for 4 h before initial heat exposure and BSO left on for the remainder of study; Curve is normalized to 12 min 45.5°C point (B45.5–10 h control surviving fraction = 0.078); (△), same protocol as 45.5°–10 h except BSO added just prior to initial heating and left on for the remainder of experiment. (b) (○), continuous exposure to 43°C; (●), 1 h exposure to 43°C, incubated at 37°C for 5 h then re-exposed to 43°C; (□), same heating protocol as 43–5 h except cells were pretreated with BSO for 4 h before initial heat treatment and BSO left on for the remainder of study; Curve is normalized to 1 h 43°C point (B43–5 h control surviving fraction = 0.095); (△), same as 43–5 h except BSO added just prior to initial heating and left on for the remainder of experiment.

(continuous heating) where survival decreased exponentially with time at 45.5°C. Pronounced thermotolerance was observed when initially heated cells were subsequently incubated at 37°C for 10 h (open circles) before re-exposure to 45.5°C. When GSH synthesis was inhibited by BSO 4 h prior to the initial heating (open squares) or immediately prior to the initial heating (open triangles) the extent of thermotolerance expressed was clearly decreased. The extent of sensitization by both GSH protocols was essentially the same. Neither protocol of GSH depletion resulted in the complete elimination of thermotolerance. Shown in Figure 1(b) are similar data using a 1 h 43°C initial exposure.

Heat stress protein (HSP) and total protein synthesis was determined for the various heating schedules described above and are shown in Figure 2 and Table II. Figure 2(a) illustrates the pattern in HSP synthesis following the 12 min 45.5°C exposure. The initial heat treatment clearly inhibited total protein synthesis (45.4–0 h, also see Table II). Recovery of total protein synthesis and HSP synthesis are shown in the 5 and 10 h post-

**Table II** Total protein synthesis for heated cells.

| *Time after 12 min 45.5°C treatment (h)* | *Total protein synthesis*[a] *(% of control)* | | | |
|---|---|---|---|---|
| | *Heat only* | *(B45.5°)* | *(45.5°B)* | *Control (no heat + BSO)* |
| 0 | 7 | ND | ND | ND |
| 5 | 31 | 10 | 26 | 70 |
| 10 | 60 | 25 | 52 | 52 |
| *Time after 1 h 43°C treatment (hr)* | *Total protein synthesis*[a] *(% of control)* | | | |
| | *Heat only* | *(B43°)* | *(43°B)* | *Control (no heat + BSO* |
| 0 | 11 | 8.5 | ND | ND |
| 2.5 | 47 | 19 | ND | ND |
| 5 | 80 | 37 | 27 | 59 |

[a]Total protein synthesis as measured by $^{35}$S-methionine incorporation and liquid scintillation counting.
ND = Not Determined.

**Figure 2** Autoradiogram of an SDS-polyacrylamide slab gel of $^{35}S$ proteins extracted from control and heat treated V79 cells. Molecular weight standards (Kd) are shown in left-hand margins and molecular weights (Kd) for specific HSP are indicated in right-hand margins. (a) Heating protocol same as shown in **Figure 1(a)**; C, control; CB–10 h represents control cells treated with 10 mM BSO for 10 h. (b) Heating protocol same as shown in **Figure 1(b)**.

heat treatment columns with HSP indicated at 70, 87, and 103 Kd. The 10 h post-heat treatment HSP pattern represents the status of the cells in this respect just prior to re-exposure at 45.5°C (see Figure 1(a)). Upon visual comparison, there are clearly decreased quantities of HSP present (compared to heat alone, 45.4–10 h) for the BSO treated cells. A more quantitative assessment of the HSP synthesis is shown by densitometer scanning studies shown in Figure 3. Pre-BSO treatment when GSH was <5% prior to heating resulted in the greatest inhibition of specific HSP (B45.5–10 h, Figure 2(a), Figure 3). BSO added immediately prior to heat treatment also results in decreased quantities of HSP that are intermediate between heat treatment alone and pre-BSO treatment. Thermotolerance development as measured by survival for both GSH depletion protocols was similar (Figure 1(a)) despite the difference in specific HSP synthesized. Since we had previously shown that BSO pretreatment or BSO added just prior to heating does not appreciably sensitize cells to continuous heating at 45.5°C (Mitchell & Russo, 1983), the sensitization observed in the present study in regard to thermotolerance is not a result of inherent sensitization by BSO. It should be noted that prolonged BSO treatment of control cultures partially inhibited total protein synthesis as shown in Table II. The inhibition observed was gradual with time and may have resulted as an indirect cellular effect of prolonged GSH deprivation (Mitchell *et al.*, 1983). Quantitative densitometry measurements showed that a non-specific protein such as actin (43 Kd) was inhibited by BSO treatment to the same extent as the specific HSP studied (data not shown). This probably reflects an indirect effect resulting from prolonged GSH depletion, however we cannot rule out direct toxicity from BSO.

Similar data are shown in Figure 2(b) and Table II for the 43°C protocol. Qualitatively, the overall results are similar to the 45.5°C schedule with the exception that the 87 Kd HSP was not observed.

### Discussion

The results of this study demonstrate that GSH depletion and inhibition of GSH synthesis by BSO interferes with cellular recovery processes following acute heat exposures of 43°C and 45.5°C. When cells were depleted of GSH to <5% of control or GSH synthesis prevented just prior to acute heat exposure, thermotolerance induction was decreased; likewise, the relative quantities of specific HSP were also decreased. These observations suggest that GSH, or the indirect effects associated with GSH depletion, at least in part, may be of importance in

**Figure 3** Relative quantities of HSP 70, 87, 103 Kd as a function of time after the initial 12 min 45.5°C heat treatment. Values of these HSP were determined by densitometry measurements from gel autoradiograms and compared to control bars at the same mol. wt. The various conditions cited correspond to conditions described in **Figure 1(a)**.

the initial cellular responses to thermal stress. GSH has long been known as a central compound in maintaining the cellular redox state (Chance, 1979), in protection from oxidation stress brought about by oxygen metabolism (Chance, 1979), and in detoxification of toxic compounds through reactions with GSH transferase (Jakoby, 1980). The maintenance of the necessary ratio of reduced to oxidized glutathione has been reported to be involved in regulation of protein translation (Kosower *et al.*, 1972). Additionally, cysteine metabolism has been linked with glutathione in that GSH may be an intracellular storage depot for the more labile and toxic cysteine (Cooper, 1983; Meister & Anderson, 1983). BSO was selected in these studies to deplete GSH by virtue of its selectivity; however, the indirect effects of prolonged depletion of an important compound such as GSH may afford yet other stresses for which the cell must adapt. For example, while the plating efficiency for unheated GSH depleted cells remained virtually unchanged over 10 h, total protein synthesis was decreased (see Table II). Moreover, since cysteine is a necessary amino acid for protein synthesis, the decrease in total protein synthesis as seen by monitoring actin synthesis can be construed as an indirect effect of GSH depletion. Nevertheless, this does not obviate but rather emphasizes the importance of GSH in cellular regulation.

While thermotolerance development was decreased for GSH depleted cells, it was not completely inhibited. This may imply that GSH asserts a partial role in maintenance of cellular integrity required for the development of thermal tolerance induction. Another interpretation is that despite our lack of detection of GSH, different cellular pools of GSH might only be partially or more slowly depleted by BSO, and hence the partial thermal tolerance response. We are currently pursuing the issue of selective pools of GSH and how these may effect thermal tolerance induction. The possibility of low levels of endogenous HSP being present prior to heating has been reported (Anderson, 1982). This possibility has not been ruled out for V79 Chinese hamster cells. Other methods of induction of thermal tolerance and/or HSP synthesis such as ethanol (Li & Hahn, 1982; Li, 1983) or arsenite (Landry and Chretien, 1983) have been shown to increase intracellular levels of GSH (Mitchell *et al.*, 1983; Henle *et al.*, 1983). Unfortunately, each chemical may have multiple effects that may negate the final outcome, i.e., the induction of thermal tolerance. This is clearly seen with arsenite pretreatment of cells; HSP synthesis is seen without thermal tolerance induction (Landry & Chretien; 1983). The underlying mechanism(s) for HSP synthesis and/or thermotolerance expression are not well understood. Considerable data are accumulating which supports the premise that cellular oxidative stress is important in HSP induction (Ashburner & Bonner, 1979; Mitchell *et al.*, 1983). Consequently, GSH metabolism should play a major role in cellular thermal adaptation. The data presented here support this concept and further studies are underway to clarify the role of oxidative stress during hyperthermia.

**References**

ANDERSON, N.L., GIOMETTI, C.S., GEMMELL, M.A., NANCE, S.L. & ANDERSON, N.G. (1982). A two-dimensional electrophoretic analysis of the heat-shock-induced proteins of human cells. *Clin. Chem.*, **28**, 1984.

ASHBURNER, M. & BONNER, J.J. (1979). The induction of gene activity in Drosophila by heat shock. *Cell*, **17**, 241.

CHANCE, B., SIES, H. & BOVERIS, S. (1979). Hydroperoxide metabolism in mammalian organs. *Physiol. Rev.*, **59**, 527.

COOPER, A.J.L. (1983). Biochemistry of sulfur-containing amino acids. *Ann. Rev. Biochem.*, **52**, 187.

DETHMERS, J.K. & MEISTER, A. (1981). Glutathione export by human lymphoid cells: Depletion of glutathione by inhibition of its synthesis decreases export and increases sensitivity to irradiation. *Proc. Natl Acad. Sci.*, **78**, 7492.

GRIFFITH, O.W. & MEISTER, A. (1979). Potent and specific inhibition of glutathione synthesis by buthionine sulfoximine (S-*n*-butyl homocysteine sulfoximine). *J. Biol. Chem.*, **254**, 7558.

HAHN, G.M. & LI, G.C. (1982). Thermotolerance and heat shock proteins in mammalian cells. *Radiat. Res.*, **92**, 452.

HENLE, K.J., NAGLE, W.A. & MOSS, A.J., JR. (1983). Development of thermotolerance following the oxidation of cellular glutathione. *Radiat. Res.*, **94**, 584.

JAKOBY, W.B. & HABIG, W.H. (1980). Glutathione transferases. In *Enzymatic Basis of Detoxification* (Ed. Jakoby). New York: Academic Press, p. 63.

KOSOWER, N.S., VANDERHOFF, G.A. & KOSOWER, E.M. (1972). Glutathione III. The effects of glutathione disulfide on initiation of protein synthesis. *Biochim. Biophys. Acta*, **272**, 623.

LAEMMLI, U.K. (1970). Cleavage of structural proteins during the assembly of the head of bacteriophage T4. *Nature*, **227**, 680.

LANDRY, J. & CHRETIEN, P. (1983). Relationship between hyperthermia-induced heat-shock proteins and thermotolerance in Morris hepatoma cells. *Can. J. Biochem. Cell. Biol.*, **61**, 428.

LI, G.C. (1983). Induction of thermotolerance and enhanced heat shock protein synthesis in Chinese hamster fibroblasts by sodium arsenite and by ethanol. *J. Cell. Phys.*, **115**, 116.

LI, G.C. & HAHN, G.M. (1982). Thermotolerance and heat shock proteins in mammalian cells. *Radiat. Res.*, **92**, 452.

LI, G.C. & WEBB, Z. (1982). Correlation between synthesis of heat shock proteins and development of thermotolerance in Chinese hamster fibroblasts. *Proc. Natl Acad. Sci.*, **79**, 3218.

MEISTER, A. & ANDERSON, M.E. (1983). Glutathione. *Ann. Rev. Biochem.*, **52**, 711.

MERRIL, C.R., GOLDMAN, D., SEDMAN, S.A. & EBERT, M.H. (1981). Ultra-sensitive stain for protein in polyacrylamide gels show regional variation in cerebrospinal fluid protein. *Science*, **211**, 1437.

MITCHELL, J.B., RUSSO, A., KINSELLA, T.J. & GLATSTEIN, E. (1983). Glutathione elevation during thermotolerance induction and thermosensitization by glutathione depletion. *Cancer Res.*, **43**, 987.

MITCHELL, J.B. & RUSSO, A. (1983). Thiols, thiol depletion, and thermosensitivity. *Radiat. Res.*, **95**, 471.

TANGUAY, R.M. (1983). Genetic regulation during heat shock and function of heat-shock proteins: A review. *Can. J. Biochem. Cell Biol.*, **61**, 387.

TIETZ, F. (1969). Enzymic method for quantitative determination of nanogram amounts of total and oxidized glutathione: applications to mammalian blood and other tissues. *Anal. Biochem.*, **27**, 502.